# Variability and trends in corticosteroid use by male United States participants with Duchenne muscular dystrophy in the Duchenne Registry

**DOI:** 10.1186/s12883-019-1304-8

**Published:** 2019-05-02

**Authors:** Leslie Cowen, Maria Mancini, Ann Martin, Ann Lucas, Joanne M. Donovan

**Affiliations:** 1 Catabasis Pharmaceuticals, One Kendall Square, Cambridge, MA 02139 USA; 2 Parent Project Muscular Dystrophy, Hackensack, NJ USA; 3 Present address: Sanofi Genzyme, Cambridge, MA USA

**Keywords:** Duchenne muscular dystrophy, DuchenneConnect, The Duchenne Registry, Rare disease registries, Corticosteroids

## Abstract

**Background:**

Treatment options for Duchenne muscular dystrophy remain limited, although consensus treatment guidelines recommend corticosteroid use.

**Methods:**

This retrospective analysis assessed corticosteroid use in ambulatory and nonambulatory US males with Duchenne, age 35 and under, or Becker muscular dystrophy, who enrolled in The Duchenne Registry from 2007 to 2016 (formerly DuchenneConnect).

**Results:**

The mean (SD) age of corticosteroid use initiation was 5.9 (2.5) years, and deflazacort use (54%) was slightly more common than prednisone/prednisolone (46%). Among all responses from those with Duchenne, 63% were currently on corticosteroids, 12% were no longer on corticosteroids, and 25% had never been on corticosteroids. Among those who were nonambulatory, 49% were currently on corticosteroids, 28% had discontinued corticosteroids, and 23% had never used corticosteroids. Primary reasons for never initiating therapy were that corticosteroids were not prescribed or recommended and concerns about side effects. Corticosteroid use was maximal at age 8 (84% on corticosteroids) and gradually declined from age 10 to 19. The primary reasons for corticosteroid discontinuation were problems with side effects (65%) or not enough benefit (28%). Average doses of corticosteroids were below recommended doses. In the 159 responses with Becker muscular dystrophy, 20% were currently using corticosteroids.

**Conclusions:**

Recognizing the self-selected nature of participation, it appears that a considerable proportion of US participants registered with The Duchenne Registry were either not on corticosteroids or not on recommended doses despite consensus recommendations. Side effects were a consideration in initiating and discontinuing treatment.

These data reinforce the need for additional treatment options for those affected by Duchenne.

## Background

Duchenne muscular dystrophy (Duchenne) is the most common genetic neuromuscular disease in children. Mutations in the X-linked dystrophin gene cause complete absence of dystrophin protein in skeletal, cardiac and respiratory muscle. In the absence of dystrophin, stress from repeated muscle contractions damages the sarcolemma and results in repeated cycles of inflammation, cellular degeneration and progressively failing muscle regeneration [1, 2]. Birth prevalence is approximately 1 in 5000 newborn males [3]. Becker muscular dystrophy (Becker) is a milder dystrophy caused by partial absence of dystrophin and is less common than Duchenne with a later onset and slower course [4].

Although onset of Duchenne pathology can be observed in infancy [5], clinical symptoms typically manifest between 3 to 5 years of age with muscle weakness and wasting. Progressive lower limb muscle weakness results in loss of ambulation in adolescence, while respiratory muscle weakness leads to respiratory insufficiency requiring ventilation support beginning in late teens/early adulthood [6]. The use of ventilation support and glucocorticoids is associated with delayed loss of functional milestones and increase in life span [7] by several years from the previous median life expectancy of 19 years [8]. However, Duchenne is still associated with early mortality, most often from cardiac or respiratory failure, typically before 30 years of age [9].

Treatment options for Duchenne are limited. Eteplirsen (EXONDYS 51®) is an approved exon–skipping drug that targets exon 51 in the dystrophin gene and is appropriate for approximately 13% of patients with Duchenne [10]. Glucocorticoid therapy has been shown to have multiple benefits, including delaying loss of muscle function, loss of ambulation, onset of scoliosis, and respirator dependence, and improving strength and mobility [11–13]. A retrospective study of 97 patients with Duchenne reported improved functional outcomes with manageable adverse effects following 8.5 years of daily corticosteroid therapy [14]. A 10-year longitudinal study that followed males with Duchenne, with and without glucocorticoid therapy, reported that glucocorticoid treatment was associated with reduced risk of losing clinically meaningful mobility and upper limb disease progression milestones across the lifespan, as well as reduced risk of death [7]. However, long-term corticosteroid use can result in numerous side effects including growth inhibition, delay of puberty, weight gain, behavioral changes, long bone and vertebral fractures, Cushingoid facies and habitus, cataracts, and insulin resistance [15, 16], and current guidelines provide recommendations for management of side effects. Corticosteroid use long-term can also lead to myopathy through induction of several cellular pathways involving ubiquitin ligase (e.g., *Murf1*, *atrogin1)* and insulin-like growth factor I (IGF 1) [17, 18]. Thus, use of corticosteroids in patients with Duchenne requires diligent monitoring to ensure that both clinical benefits and side effect risks are managed appropriately [19]. The two most commonly prescribed corticosteroids in the US are prednisone and deflazacort [12], which was recently approved for Duchenne in the US (EMFLAZA®) [20]. Deflazacort has been proposed to have fewer side effects than prednisone [11, 21, 22]. Evidence-based starting dose recommendations are 0.9 mg/kg/day and 0.75 mg/kg/day (or 10 mg/kg/weekend) for deflazacort and prednisone, respectively, with dose reductions as necessary [11, 19]. However, there is considerable variability in corticosteroid prescribing patterns for patients [12, 23, 24]. The purpose of this study was to understand the landscape of corticosteroid use in ambulatory and nonambulatory patients in the United States (US) with Duchenne or Becker muscular dystrophy.

## Methods

Information entered by respondents enrolled in The Duchenne Registry was the primary data source. The Duchenne Registry, formerly known as DuchenneConnect, is a patient self-report registry and educational resource for individuals with Duchenne or Becker and carrier females [25]. The registry was established in 2007 by Parent Project Muscular Dystrophy and can be accessed at www.duchenneregistry.org. The majority (70%) of enrolled participants are from the US. Participant data are curated by coordinators, and requests for updates to participant profiles occur every 6–12 months. Participants complete survey questionnaires on up to 12 topics, including diagnosis, mobility and musculoskeletal function, respiratory and cardiac function, and therapies including corticosteroid use. Because of the self-reported nature, not all questions were completed by all participants. As a result, the total number of responses varies for different questions. Data are maintained in a HIPAA-compliant database. All participants (patients 18 years and older or parents/ custodians/legal guardians of children under the age of 18) granted permission for de-identified information shared in the registry to be provided to researchers. Only The Duchenne Registry coordinators employed by Parent Project Muscular Dystrophy have access to identifying information. Participant permission was obtained via an electronic consent process at the time of registry account creation. The Duchenne Registry and electronic consent are approved by the Geisinger Institutional Review Board (Geisinger Medical Center, Danville, PA, IRB#2014–0621).

The Duchenne Registry corticosteroid module captures information on current corticosteroid use patterns, reasons for discontinuing, and reasons for never initiating corticosteroids. Analysis of The Duchenne Registry database for information captured in the corticosteroid module was performed for US male Duchenne and Becker participants responding from October 2007 through November 2016. For participants with a diagnosis of Duchenne, only responses from males age 35 and under were included in the analysis. Selected analyses were also performed for males of any age diagnosed with Becker, and these are presented separately from the analyses for males with Duchenne.

Descriptive statistics (mean, standard deviation, median and range) were determined for participant responses. Age for steroid use refers to the age reported at the time the response was provided. If the respondent indicated that steroid use was unknown, they were not included in this analysis. For analysis by birth cohort, birth age was calculated from the age provided and the date the questionnaire was completed.

Respondents could submit additional responses at a later time point, although only a minority did (82 respondents completed the questionnaire two or three times for a total of 87 additional responses). Additional responses occurred an average of 2.7 years after the initial response, with a maximum of 9 years later. If an individual submitted two responses that captured one-time past events such as age of initiation of steroids, only the most recent response was used in analyses. For module questions regarding current corticosteroid steroid use, responses collected at different ages were included since they provided additional information on use of steroids at different ages.

## Results

### Participants

**Fig. 1:**
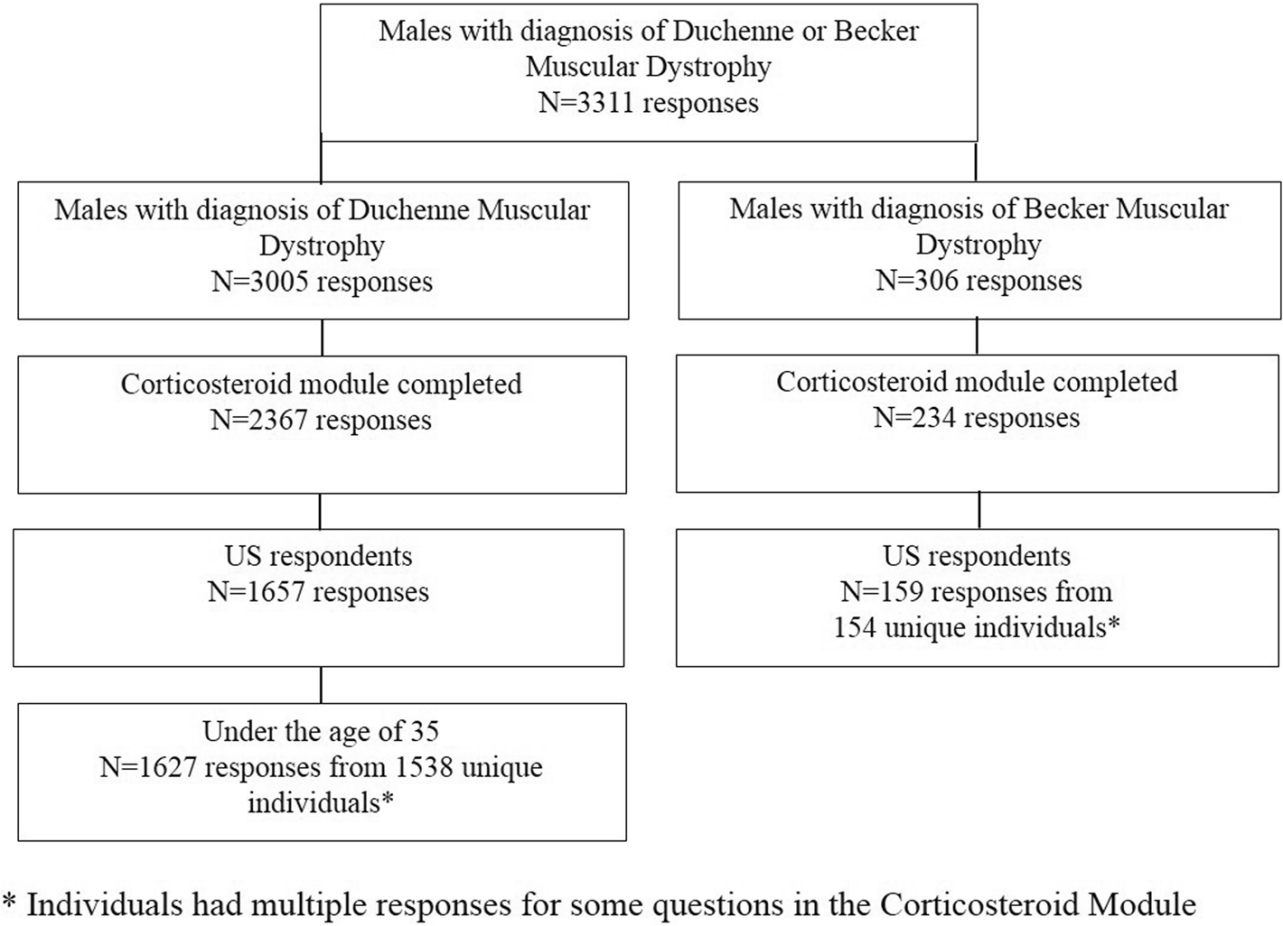
Flow Diagram for Responses in The Duchenne Registry Study for Participants with Duchenne or Becker Muscular Dystrophy

As of December 31, 2016, The Duchenne Registry had 3791 total registrants, and there were 3560 responses in which a diagnosis was reported. Of these, 3311/3560 (93.0%) were analyzed, 3005/3560 (84.4%) were responses for a diagnosis of Duchenne, and 306/3560 (8.6%) were for a diagnosis of Becker. As shown in Fig. 1, among 3311 responses from only males with a diagnosis of Duchenne or Becker, there were 2601 responses to the corticosteroid module, and 1816 of these responses were for US participants. After excluding responses from US males diagnosed with Duchenne who were over the age of 35 (*n* = 30), there were 1627 responses from 1538 individuals, and 159 responses from 154 individuals of any age diagnosed with Becker.

Table 1 shows the characteristics of the US males age 35 or under with Duchenne and US males with Becker who provided data in the corticosteroid module. Among males with Duchenne, mean age (SD) was 11.3 (6.5) years, the majority (88.9%) were less than 20 years old and Caucasian (87.2%), and most individuals were ambulatory (59.3%), with 40.0% nonambulatory. The mean age (SD) of onset for full-time use of a scooter/wheelchair was 10.8 (2.5) years. Among males with Becker, the mean age (SD) was 21.8 (16.2) years and the age distribution was broad. The majority were Caucasian (89.7%) and most individuals were ambulatory (76.4%).

**Table 1:** Characteristics of Duchenne Registry Respondents (Males with Duchenne and ≤ 35 years old) who completed the Corticosteroid Module)

Characteristic	Number of Responses and Number of Patients	
	1627 from 1538 males with Duchenne	159 from 154 males with Becker
Age (years)		
Mean (SD)	11.3 (6.5)	21.8 (16.2)
Median (1st quartile, 3rd quartile)	10 (7, 15)	17 (9, 34)
Age Category n, (%)		
≤ 4 years old	200 (12.3)	8 (5.1)
5–9 years old	544 (33.4)	35 (22.3)
10–14 years old	470 (28.9)	24 (15.3)
15–20 years old	232 (14.3)	19 (12.1)
21–24 years old	99 (6.1)	15 (9.6)
25–29 years old	54 (3.3)	9 (5.7)
30–35 years old	28 (1.7)	11 (7.0)
> 35 years old	NA	33 (21.0)
Race *n*, (%)		
Caucasian	1406 (87.2)	140 (89.7)
Asian	83 (5.1)	6 (3.8)
Other	72 (4.5)	5 (3.2)
Black	29 (1.8)	3 (1.9)
Native American	18 (1.1)	2 (1.3)
Pacific Islander	4 (< 1)	0
Ethnicity *n*, (%)		
Not Latino/Hispanic	1392 (90.2)	143
Latino/Hispanic	152 (9.8)	7
Ambulatory Status *n*, (%)	*N* = 1505	*N* = 140
Child is an infant/toddler and has not yet taken his/her first steps	11 (0.7)	0
Ambulatory	892 (59.3)	107 (76.4)
Usually/always walk without help or mobility devices	645 (42.9)	90 (64.3)
Sometimes need help from a mobility device	247 (16.4)	17 (12.1)
Nonambulatory		
Use a wheelchair/other mobility device and rarely/ never walk	602 (40.0)	33 (23.6)

### Corticosteroid use patterns

The use of corticosteroids for males with Duchenne or Becker is shown in Table 2. Among those with Duchenne, 63.0% (969/1538) reported the current use of steroids, with deflazacort use slightly greater than prednisone/prednisolone use (54.1% vs 45.9% of those currently using corticosteroids, respectively). Twenty-five percent (381/1538) of males with Duchenne had never used corticosteroids and 12.2% (188/1538) had a previous history of using corticosteroids, but were no longer using them. Among 569 nonambulatory males with Duchenne, 23.0% (131/569) had never been on steroid therapy, 48.7% (277/569) were currently using corticosteroids, and 28.3% (161/569) had previously used corticosteroids. Deflazacort and prednisone/prednisolone use among nonambulatory males was similar to use in the overall Duchenne group (47.7% vs 52.3%, respectively).

**Table 2:** Corticosteroid Use and Status in US Males with Duchenne Muscular Dystrophy under the Age of 35, and US Males with Becker Muscular Dystrophy Enrolled in The Duchenne Registry

	Duchenne			Becker
Population	All	Ambulatory^b^	Nonambulatory^b^	All
Males with Duchenne Muscular Dystrophy *n*, (%)	*N* = 1538	*N* = 845	*N* = 569	*N* = 154
Currently using corticosteroids	969 (63.0)	604 (71.5)	277 (48.7)	30 (19.5)
*Deflazacort* ^a^	*524 (54.1)* ^a^	*344 (57.0)* ^a^	*132 (47.7)* ^a^	*21 (70.0)* ^a^
*Prednisone/prednisolone* ^a^	*445 (45.9)* ^a^	*260 (43.0)* ^a^	*145 (52.3)* ^a^	*9 (30.0)* ^a^
Never used corticosteroids	381 (24.8)	223 (26.3)	131 (23.0)	115 (74.6)
Used to take corticosteroids	188 (12.2)	18 (2.1)	161 (28.3)	9 (5.8)

^a^Percentages were calculated from number of males currently using corticosteroids as the denominator

^b^Not all respondents provided ambulatory status

Among 154 males with Becker, 19.5% (30/154) currently used corticosteroids, mostly deflazacort, 5.8% (9/154) were no longer using corticosteroids, and 74.6% (115/154) had never used corticosteroids.

**Fig. 2:**
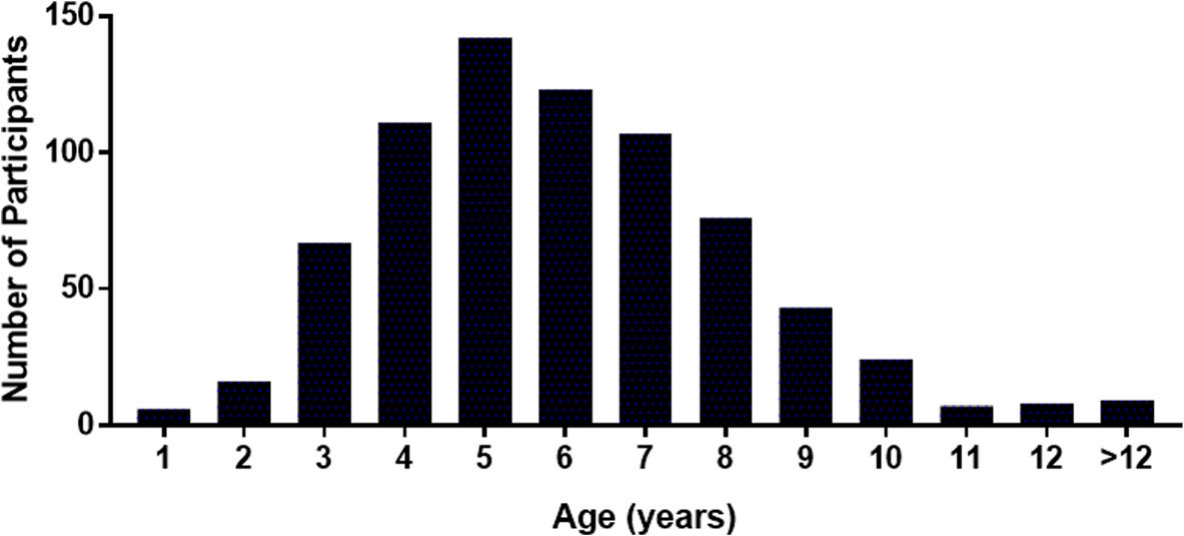
Age Distribution at Initiation of Corticosteroid therapy for Males with Duchenne Muscular Dystrophy. Unique responses for age at corticosteroid initiation were provided by 651 males with Duchenne that had ever used steroids

Unique responses for age at corticosteroid initiation were provided by 651 males with Duchenne that had ever used steroids. The age distribution at initiation of treatment is shown in Fig. 2. The modal age for treatment initiation was 5 years, with a mean (SD) of 5.9 (2.5) years. Almost all of those who were ever treated with corticosteroids (637/651, 97.8%) initiated treatment before 12 years of age.

**Fig. 3:**
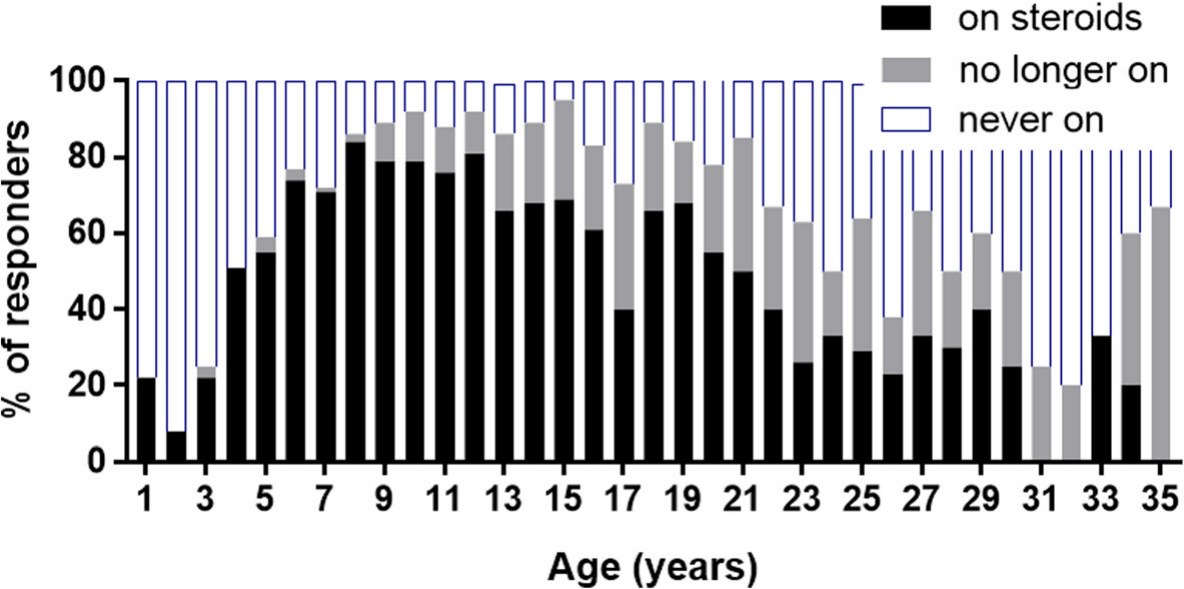
Corticosteroid Treatment Status for Males with Duchenne Muscular Dystrophy. The percentages of males with Duchenne receiving corticosteroid therapy, no longer receiving therapy, or never on corticosteroids are shown stratified by patient age

For males with Duchenne, treatment status by age is shown in Fig. 3. The highest use of corticosteroids was between 8 and 12 years of age, where at least 76.3% of males at any age were on therapy. After age 9, more than 10% had discontinued corticosteroid treatment in each age group.

**Fig. 4:**
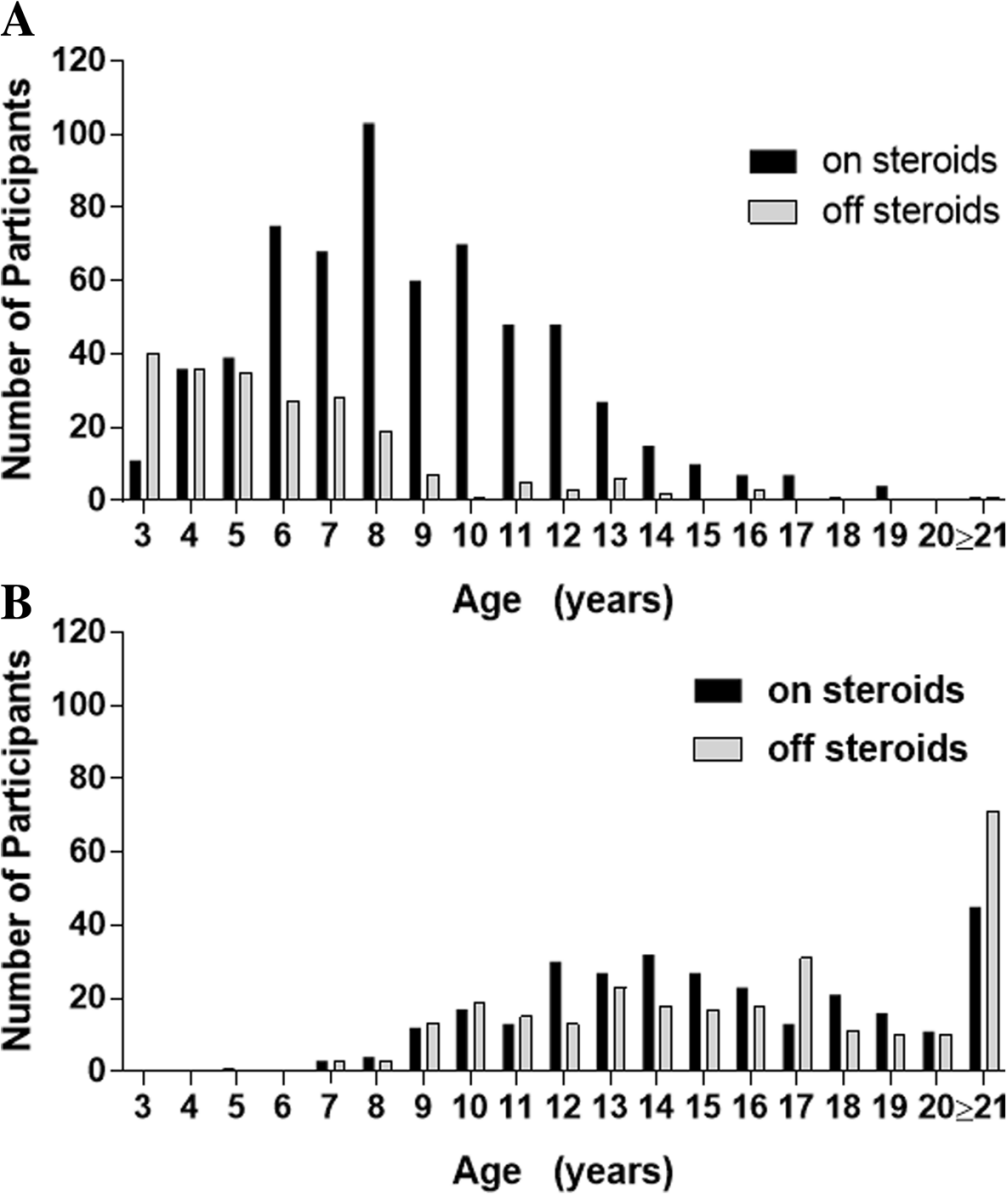
Corticosteroid Status for Males with Duchenne Muscular Dystrophy Stratified by Age. Numbers of ambulatory (**a**) and nonambulatory (**b**) patients treated with corticosteroids and not treated with corticosteroids are shown by patient age

Corticosteroid use by age for ambulatory and nonambulatory males with Duchenne is shown in Fig. 4a and b, respectively. The majority of ambulatory participants age 10 or under (67%) and over the age of 10 (89%), were using corticosteroid therapy. In contrast, among nonambulatory patients ≤ or > 10 years of age, 49 and 52%, respectively, were using corticosteroid therapy.

### Corticosteroid dose

**Fig. 5:**
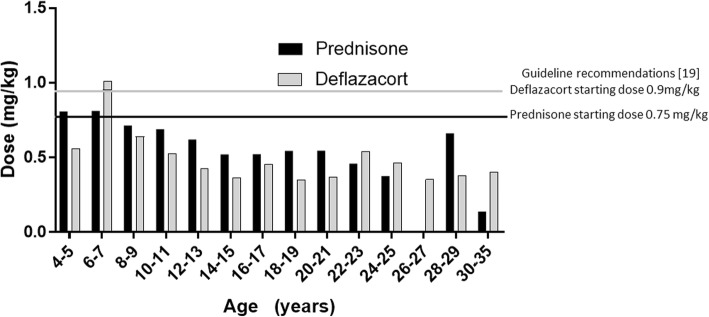
Average Corticosteroid Dose Stratified by Age Group for Males with Duchenne Muscular Dystrophy. Mean reported dose of prednisone or deflazacort used by males with Duchenne is shown for each age group. Horizontal lines indicate recommended prednisone (black line) and deflazacort (grey line) starting doses [19]

The average doses of prednisone/prednisolone and deflazacort reported by participants overall were 0.5 and 0.6 mg/kg, respectively, corresponding to 67% of the starting doses (0.75 and 0.9 mg/kg, respectively) recommended in current guidelines [19]. Average doses by participant age are shown in Fig. 5. Average doses of deflazacort tended to be higher at younger ages and declined with age.

### Corticosteroid history

Among 1246 males with Duchenne currently on corticosteroid therapy, 377 (29.7%) provided details on their history of corticosteroid use. The majority, 92.0% (347/377), had taken corticosteroids continuously and 7.9% (30/377) had taken at least one break from steroid use. As shown in Table 3, among the 46 males who discontinued corticosteroid therapy and provided reasons for discontinuation, the primary reported reason for discontinuation was problems with side effects (30/46, 65.2%), including known side effects such as weight gain, behavioral changes, osteoporosis and fractures, followed by not enough benefit (13/46, 28.3%). Among the 47 males who discontinued corticosteroid therapy, 59.6% (28/47) responded that they would not consider resuming corticosteroids even if there was new information about the long-term benefits of using corticosteroids, while 40.4% (19/47) responded they would consider resuming corticosteroids.

**Table 3:** Primary Reasons for Discontinuing or not Initiating Corticosteroid Therapy among Males with Duchenne Muscular Dystrophy

Module Query	Number (%)^a^
Reasons for Discontinuing Corticosteroid Therapy *n* (%)	*N* = 46
Problems with side effects	30 (65.2)
Not enough benefits	13 (28.3)
Did not like the use of long term medication	2 (4.3)
Other	1 (2.2)
Reasons for not Initiating Corticosteroid Therapy *n* %	*N* = 114
Worried about side effects	29 (25.4)
Doctor never prescribed/recommended	26 (22.8)
Other (starting soon, too young most common)	20 (17.5)
Worried about not getting enough benefits	7 (6.1)
Does not like the use of long term medicine	1 (0.9)
Age 3 or under	31 (27.2)

^a^A minority of respondents answered these questions

One hundred fourteen males with Duchenne who had never initiated corticosteroid therapy responded with the reasons for not starting treatment shown in Table 3. The primary reasons reported were concern over side effects (25.4%) and that corticosteroids were never prescribed/recommended (22.8%), with 27.2% being age 3 or under.

### Corticosteroid use by time period

Steroid use for different birth cohorts is shown in Table 4. Age at initiation of steroids has steadily declined over time, while the use of corticosteroids increased, and the discontinuation rate has generally fallen. Since the most recent cohort (birth years 2005 to 2009) lacks full information for age 7 and up, comparison was made for the 4 to 7-year old age range. The frequency of corticosteroid use was similar for the birth cohort 2000–04 and 2005–09.

**Table 4:** Corticosteroid Use by Birth Year for Males with Duchenne Muscular Dystrophy

Birth Interval (years)	Age at initiation of steroids (years, median, 1st and 3rd quartiles)^a^	N	On steroids %, *n*	Discontinued %, *n*	Never on steroids %, *n*	Age 4 to 7 not on steroids^b^	
1980–89	9 (7, 17)	87	20.7% 18	31.0% 27	48.3% 42	NA	NA
1990–94	8 (6, 10)	138	42.8% 59	29.7% 41	27.5% 38	NA	NA
1995–99	7 (6, 8)	316	66.5% 210	21.2% 67	12.3% 39	NA	NA
2000–04	6 (4, 8)	449	77.7% 349	10.5% 47	11.8% 53	67.6%	46/68
2005–09^c^	5 (4, 6)	418	75.6% 316	3.3% 14	21.1% 88	69.6%	156/224

^a^For those who provided age at initiation (overall 60.2%, 692/1148)

^b^Only available for cohorts in which there were registrants in this age range (i.e., after birth year 2000)

^c^Youngest members of this cohort were 7 years of age

*NA* = not available

## Discussion

Glucocorticoid therapy is considered the standard of care for Duchenne [11], although variations in prescribing practices indicate uncertainty regarding when treatment should be initiated, the risk/benefit assessment of long-term therapy, dosing regimens, and when treatment should stop [24]. A recent Cochrane review of published studies concluded from randomized clinical trials that corticosteroid therapy improves strength and function with acceptable tolerability up to 6 months [26]. Deflazacort and prednisone treatment for 12 weeks at the recommended doses of 0.9 mg/kg/day and 0.75 mg/kg/day, respectively, demonstrated similar benefits in muscle strength and similar adverse effect profiles (including Cushingoid appearance, increased weight, erythema, and hirsutism) compared to placebo [21], with the limitation that conclusions on long-term corticosteroid treatment are drawn from non-randomized studies [26]. A retrospective study of 97 patients with Duchenne reported improved functional outcomes with manageable adverse effects following 8.5 years of daily corticosteroid therapy [14]. Nonetheless, management of the patient with long-term corticosteroid therapy while minimizing side effects is challenging [27]. An ongoing clinical trial in five countries will attempt to standardize corticosteroid dosing and management for patients with Duchenne [23].

Analysis of corticosteroid use by US patients registered with The Duchenne Registry reaffirms the uncertainties surrounding corticosteroid treatment regimens. Almost 25% of participants with Duchenne from ages 4 to 35 had never used corticosteroids, even though the American Academy of Neurology recommends corticosteroids for improving strength and pulmonary function [11]. The primary reasons for not using corticosteroids were that they were not prescribed/recommended, as well as concerns over side effects. It should be noted that older patients in the registry were managed prior to the time when treatment guidelines strongly recommended steroid use; however, the majority (75%) of participating registrants were younger than 15 years of age.

Among participants with Becker, a considerable minority (25.3%) had used corticosteroids. Although there are case reports suggesting that some patients with Becker may benefit from corticosteroid therapy [28, 29], risks of long-term corticosteroid use must be weighed against uncertainties of benefit in this population. It is also possible that some patients diagnosed with Becker that were prescribed corticosteroids may have been thought to have Duchenne or intermediate dystrophinopathy.

Corticosteroid use was highest in participants with Duchenne between the ages of 6 and 12, with a modal age at initiation of corticosteroid use of 5 years. Guidelines on when glucocorticoid therapy should be initiated in ambulatory patients with Duchenne recommend initiation prior to substantial decline in mobility [19]. For US registrants that were ambulatory, corticosteroid use peaked at age 8 years. Decisions on when to initiate corticosteroid therapy are typically based on an individual’s age, functional state, and pre-existing risk factors for potential corticosteroid adverse effects. In contrast, corticosteroid use in younger participants between 4 and 8 years of age did not increase notably. These data reinforce that there are concerns/uncertainty among patients and prescribers regarding side effects and efficacy.

After the age of 9, at least 10% of respondents had discontinued steroids. The primary reasons reported for discontinuation included problems with side effects and not enough benefit. For participants who discontinued corticosteroid therapy, the majority of participants would not consider resuming corticosteroids even if there was new information about long-term benefits. While the reason for not considering resuming corticosteroids was not captured, problems with side effects are likely contributing factors. Discontinuations may also reflect the uncertainty surrounding both long-term use of corticosteroids and whether corticosteroid therapy should be maintained once ambulation decreases. Mean age at loss of ambulation for participants with Duchenne was between 10 and 11 years of age. Corticosteroid use was highest among nonambulatory participants between the ages of 12 and 18. While the case is made for continuation of glucocorticoids after loss of ambulation in order to preserve upper limb strength and respiratory function and reduce the need for spinal surgery due to scoliosis [30, 31], the risk/benefit for individual patients is a consideration.

Deflazacort use was slightly greater than use of prednisone/prednisolone, which is notable since deflazacort was not approved in the US for use in Duchenne until after these data were collected [20]. The substantial use of deflazacort may reflect a selection bias in that parents/patients that are registry participants are likely to be better informed and were more likely to be able to request and obtain deflazacort, given published evidence that deflazacort may be associated with greater preservation of muscle function [22]. Clinical trials also indicate that deflazacort may result in less weight gain than prednisone but is associated with decreased stature and more asymptomatic cataracts [11, 21]. A randomized controlled trial assessing corticosteroid therapy for Duchenne is ongoing [23].

In 2016, the American Academy of Neurology (AAN) recommended a starting dose of prednisone at 0.75 mg/kg/day (or 10 mg/kg/weekend) [11] and the approved label dose of deflazacort is 0.9 mg/kg/day [20]. Interestingly, the doses of prednisone and deflazacort reported by participants in The Duchenne Registry varied considerably by age (with trends for decreases in older patients), and the average doses were 67% of the recommended doses. These results are similar to results reported in a Duchenne natural history study where average doses of prednisone/prednisolone and deflazacort where 75 and 83%, respectively, of the recommended doses [12]. It is important to note that The Duchenne Registry does not capture specific doses of corticosteroids prescribed by practitioners, but rather the reported actual dose, which may account for some of the variability.

Patient and family participation in The Duchenne Registry is extremely valuable in order to further clinical development of new treatments for Duchenne. The Duchenne Registry is the largest US-based patient report registry and can provide important natural history data that can inform practitioners and researchers regarding trends in patient care and reasons why patients and families make treatment decisions. Inherent limitations to registry-based data analysis include that entries are based on patient/caregiver reporting and may have missing or inconsistent data which may confound results. The Duchenne Registry participants represent approximately 14% of individuals with Duchenne in the US, and systematic biases among participants prevents generalization of findings to all patients. In addition, The Duchenne Registry registrants likely represent a cohort of well-informed and motivated patients. However, the results on corticosteroid use patterns are consistent with population-based studies of corticosteroid use in males with Duchenne [32, 33]. In a retrospective study comparing males born 1982 to 1986 with males born 1997 to 2001, steroid use increased by 20% and mean age at steroid initiation decreased by year [32]. An international, center-based longitudinal study begun in 2006 assessed glucocorticoid treatment status for males with Duchenne and found that, among 340 males between the ages of 2 and 28, 62% were on corticosteroid therapy, 14% were no longer on therapy, and 24% had never been on therapy [33] at baseline. Our results in a registry US population five times the size of the 2006 study demonstrated almost identical percentages of males who were on therapy (62%), had a history of therapy (12%), and who were therapy naïve (24%).

## Conclusions

Glucocorticoids have a range of adverse side effects that influence patient initiation and continuation with therapy, and a considerable proportion of patients with Duchenne registered with The Duchenne Registry in the US are not on recommended glucocorticoid therapy. Analysis of The Duchenne Registry data reinforces the need for safe and effective treatment options for those affected by Duchenne.

## Abbreviations

AAN: American Academy of Neurology
HIPAA: Health Insurance Portability and Accountability Act
IGF 1: insulin-like growth factor type 1
*Murf1*: muscle ring-finger protein-1 gene, a ubiquitin ligase
SD: standard deviation
US: United States
